# OLDER ADULTS WITH DISABILITIES AS CORESEARCHERS IN COMMUNITY-ENGAGED RESEARCH ON MOBILITY AND ACCESS

**DOI:** 10.1093/geroni/igae098.2020

**Published:** 2024-12-31

**Authors:** Atiya Mahmood, Niloofar Hedayati, Rojan Nasiri, Sogol Haji Hosseini, Sepehr Pandsheno

**Affiliations:** Simon Fraser University, Vancouver, British Columbia, Canada; Simon Fraser University, Vancouver, British Columbia, Canada; Simon Fraser University, Vancouver, British Columbia, Canada; Simon Fraser University, Vancouver, British Columbia, Canada; Simon Fraser University, Vancouver, British Columbia, Canada

## Abstract

Neighbourhood access and participation opportunity significantly impacts the health, social inclusion, and overall wellbeing of older adults especially those with mobility, sensory and cognitive disabilities. Stakeholders Walkability/Wheelability Audit in Neighbourhoods (SWAN) is a community engaged mixed method project where user-led audits are conducted in neighbourhoods complemented by semi-structured interviews to evaluate the role of built environment on mobility, access and participation of older adults and people with disabilities across five cities within Metro Vancouver, Canada. Using a community engaged lens starting from initial engagement to more advanced collaboration, this project involves various community stakeholders in different capacities fostering a two-way exchange of information between researchers and community members. Study participants as coresearchers participate in research tool adaptation, data collection, analysis and knowledge mobilization. Additionally, city staff members partner with the research team to identify key areas in cities for data collection, collaborate in knowledge mobilization activities and the development of complementary implementation projects. The SWAN project is part of a larger partnership project titled, “Mobility, Access and Participation” (MAP). Preliminary findings from this project underscores neighbourhood accessibility as it relates to functionality, safety, appearance, supportive features and social engagement opportunities for older adults with disabilities while highlighting some distinct challenges across different types of disabilities. Insights from the SWAN project has the potential inform both academic scholarship and local government policy around mobility, access and social. Findings underscores the importance of community engaged research in informing programmatic and policy changes using a social equity lens.

